# Spinal Metastases in Diffuse Intrinsic Pontine Glioma: A Rare Presentation of Rapid Neurological Decline

**DOI:** 10.7759/cureus.81289

**Published:** 2025-03-27

**Authors:** Grace E Markey, Ramesh Boggula, Ahmad Hammoud, Steven R Miller

**Affiliations:** 1 Department of Oncology, Wayne State University School of Medicine, Detroit, USA

**Keywords:** brainstem tumor, conformal radiation therapy, craniospinal irradiation (csi), diffuse intrinsic pontine glioma, salvage radiation

## Abstract

A nine-year-old male patient with a history of diffuse intrinsic pontine glioma (DIPG) presented with an episode of acute neurological deterioration approximately 18 months following completion of a primary course of radiation therapy to the brainstem for DIPG. His symptoms at that time included weakness in the lower extremities, urinary incontinence, and respiratory failure. Magnetic resonance imaging of the brain, cervical, thoracic, and lumbar spine revealed progression of the disease with metastatic deposits involving the craniospinal axis. He subsequently underwent a course of salvage radiation therapy to the craniospinal axis. This case highlights a rare but severe manifestation of metastatic DIPG with spinal involvement.

## Introduction

Diffuse intrinsic pontine glioma (DIPG) is a rare and highly aggressive brainstem tumor predominantly affecting children aged 5 to 10 years, with an estimated incidence of about 200-300 cases annually in the United States. It originates in the pons, a critical region of the brainstem responsible for essential functions such as breathing, balance, and motor control. DIPG is characterized by its infiltrative nature, making surgical resection unfeasible. Moreover, the tumor is resistant to chemotherapy due to the presence of the blood-brain barrier (BBB) and the lack of effective systemic agents. The disease is characterized by rapid progression and a poor prognosis, with most patients surviving less than a year [1]. Diagnosis is primarily based on magnetic resonance imaging (MRI), and treatment typically involves radiation therapy. Despite advances in various treatment strategies, including radiation, chemotherapy, and experimental therapies, survival rates have shown minimal improvement, with one-year, two-year, and three-year survival at 41%, 15.3%, and 7.3%, respectively [2,3].

The pathophysiology of DIPG is primarily driven by mutations in histone H3 genes, particularly the H3K27M mutation, which alters chromatin regulation and contributes to tumor aggressiveness. Radiographically, DIPG is identified on MRI as a T2-hyperintense diffusely infiltrative lesion within the pons, typically without contrast enhancement. (In a T2-weighted MRI image, cerebrospinal fluid (CSF) usually appears bright white, while gray matter is a lighter shade of gray. Both benign and malignant tumors can cause T2 MRI signal abnormalities.)

Diagnosis is mainly imaging-based, though a biopsy may be performed in select cases to confirm molecular markers and guide targeted therapies [2,3]. DIPG typically exhibits a locally invasive growth pattern, with tumor cells infiltrating the surrounding brainstem structures. Distant metastasis is rare and generally confined to the brainstem and adjacent brain parenchyma. However, spinal metastases, though infrequent, represent a severe and often overlooked manifestation of this malignancy [4]. Limited case reports in the literature describe such metastatic spread, highlighting the rarity of this progression and its potential to complicate clinical management. Diagnosing spinal involvement remains a significant challenge due to the overlapping clinical presentation with other conditions, often delaying timely diagnosis and intervention.

Treatment for DIPG remains palliative, with external beam radiotherapy serving as the standard of care. A total dose of 5040 to 5400 centigray (cGy) is typically administered in daily fractions of 180-200 cGy to the brainstem lesion with a margin. Target volumes are delineated using a T2 MRI, which defines the gross tumor volume (GTV). The MRI is then fused with the computed tomography (CT) simulation scan for treatment planning. The planning target volume (PTV) consists of a 1 cm margin around the GTV. Despite various studies exploring alternative fractionation methods, such as hyper-fractionated and hypo-fractionated radiotherapy, none have shown a significant improvement in survival outcomes [5]. Overall, DIPG continues to pose a therapeutic challenge, with minimal advancements in treatment options over recent decades.

In this case report, we present a pediatric patient with DIPG who experienced aggressive disease progression with rare spinal metastases. This case contributes to the growing understanding of DIPG’s metastatic potential and underscores the need for improved diagnostic, monitoring, and management strategies. By highlighting this unusual presentation, we aim to enhance awareness of metastatic DIPG and advocate for further research into more effective treatment approaches.

## Case presentation

An eight-year-old boy was initially noted by his mother to have slurred speech, drooling from the mouth, and right-hand weakness. He was initially seen in the emergency department. On examination, there were mild dysarthria, right upper extremity weakness (4/5 strength), and slightly decreased coordination on finger-to-nose testing. Cranial nerve evaluation revealed no deficits in extraocular movements or facial symmetry. Deep tendon reflexes in the upper extremities were normal, and no sensory loss was reported.

**Figure 1:**
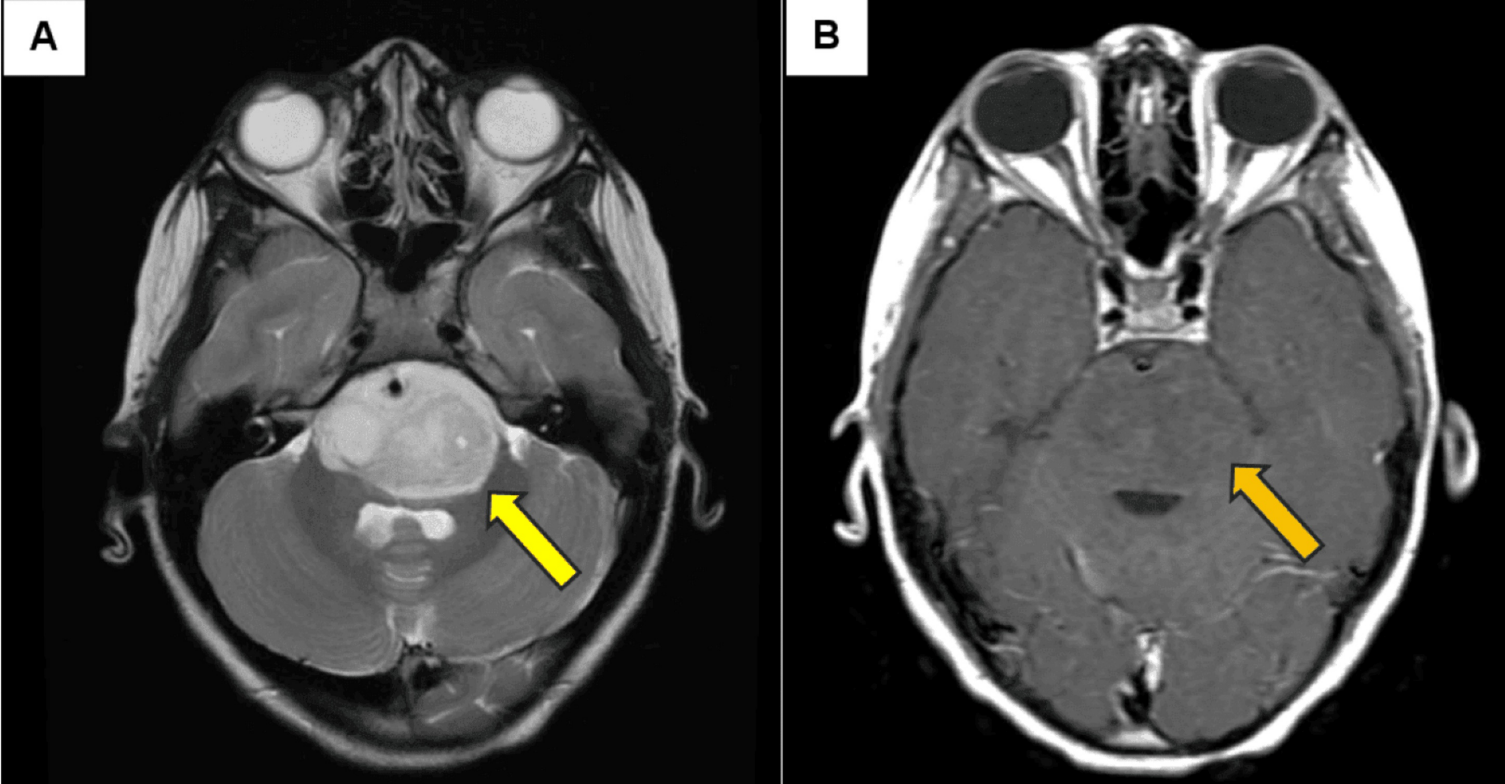
Axial T2 MRI showing a hyperintense brainstem lesion (A: yellow arrow) and T1 MRI displaying a subtle hypointense brainstem lesion (B: orange arrow). These MRI findings are consistent with a brainstem glioma.

A brain CT scan revealed a lesion involving the brainstem. A subsequent brain MRI revealed an expansile 4.3 x 2.2 x 4.6 cm heterogeneous T2 hyperintense T1 hypointense intra-axial mass lesion with an epicenter in the pons. (T2 MRI sequences highlight variations in water content and other tissue characteristics. T1 images appear bright (hyperintense) when imaging fatty tissue, and an abnormally low signal on T1 images frequently indicates a pathological process such as trauma, infection, or cancer.) These T1 and T2 findings on MRI were consistent with an underlying pediatric diffuse midline glioma or DIPG (Figure 1). 

**Figure 2:**
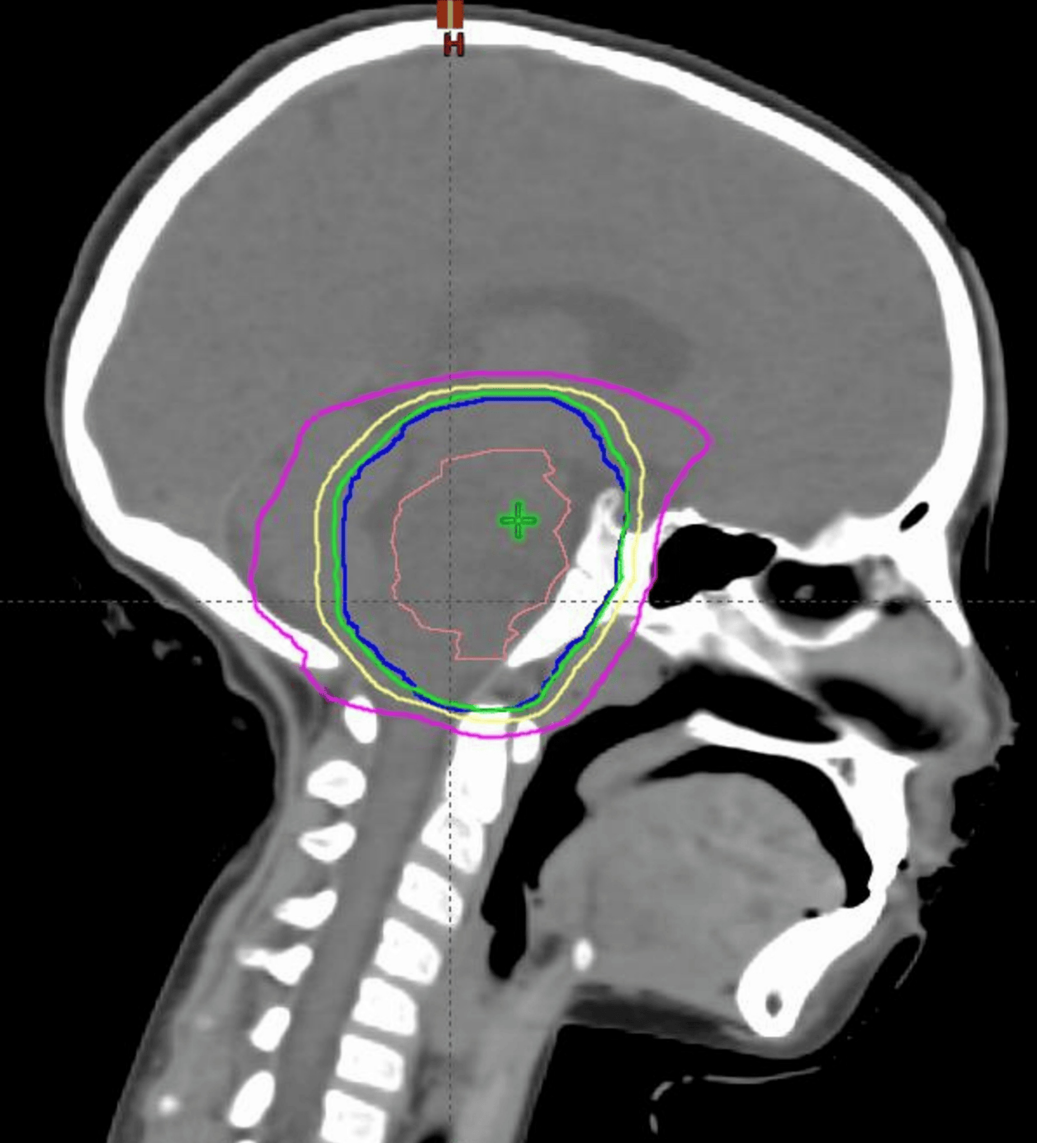
Sagittal view of the radiation therapy treatment plan to the brainstem. Gross tumor volume (GTV) is the brown contour; planning target volume (PTV) is the dark blue contour; green contour is the 95% isodose line; yellow contour is the 80% isodose line; magenta contour is the 50% isodose line

He underwent a course of definitive radiotherapy to the brainstem lesion. He received a total dose of 5040 cGy in 28 fractions to the brainstem lesion plus a 1 cm margin using intensity-modulated radiation therapy (IMRT) (Figure 2). 

Eighteen months after the completion of radiation treatment, he began experiencing increasing lower extremity pain and difficulty ambulating. He had been discharged from a prior emergency department visit after transient symptom improvement. Over the following weeks, his symptoms progressed, including severe bilateral lower extremity pain, inability to ambulate, refusal to move his left lower extremity, abdominal pain, urinary incontinence, and constipation for over a week. On the day of presentation, he developed worsening neurological decline, including lower extremity weakness, urinary incontinence, and respiratory failure, requiring resuscitation and subsequent admission to the pediatric intensive care unit (PICU).

Upon admission to the PICU, he was intubated due to respiratory distress. Neurological examination at that time revealed equal, round, and reactive pupils with a symmetric facial appearance. He withdrew his upper extremities to noxious stimuli but exhibited no motor response in the lower extremities. The biceps reflexes were preserved bilaterally, while patellar reflexes were absent.

**Figure 3:**
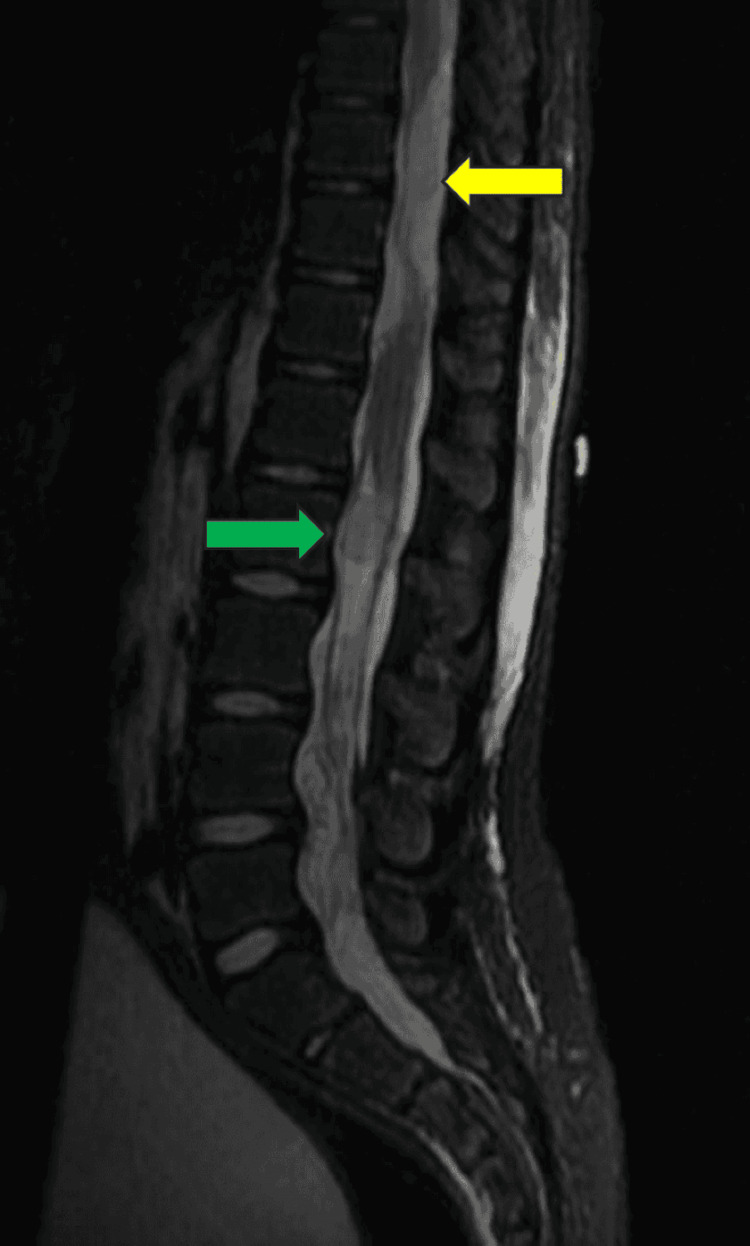
Sagittal T2 MRI with the yellow arrow pointing to the hyperintense extramedullary tumor involvement of the thoracic and lumbar spine and a green arrow pointing to intramedullary involvement of the lumbar spine with metastatic disease. The spinal cord is hypointense in this image.

**Figure 4:**
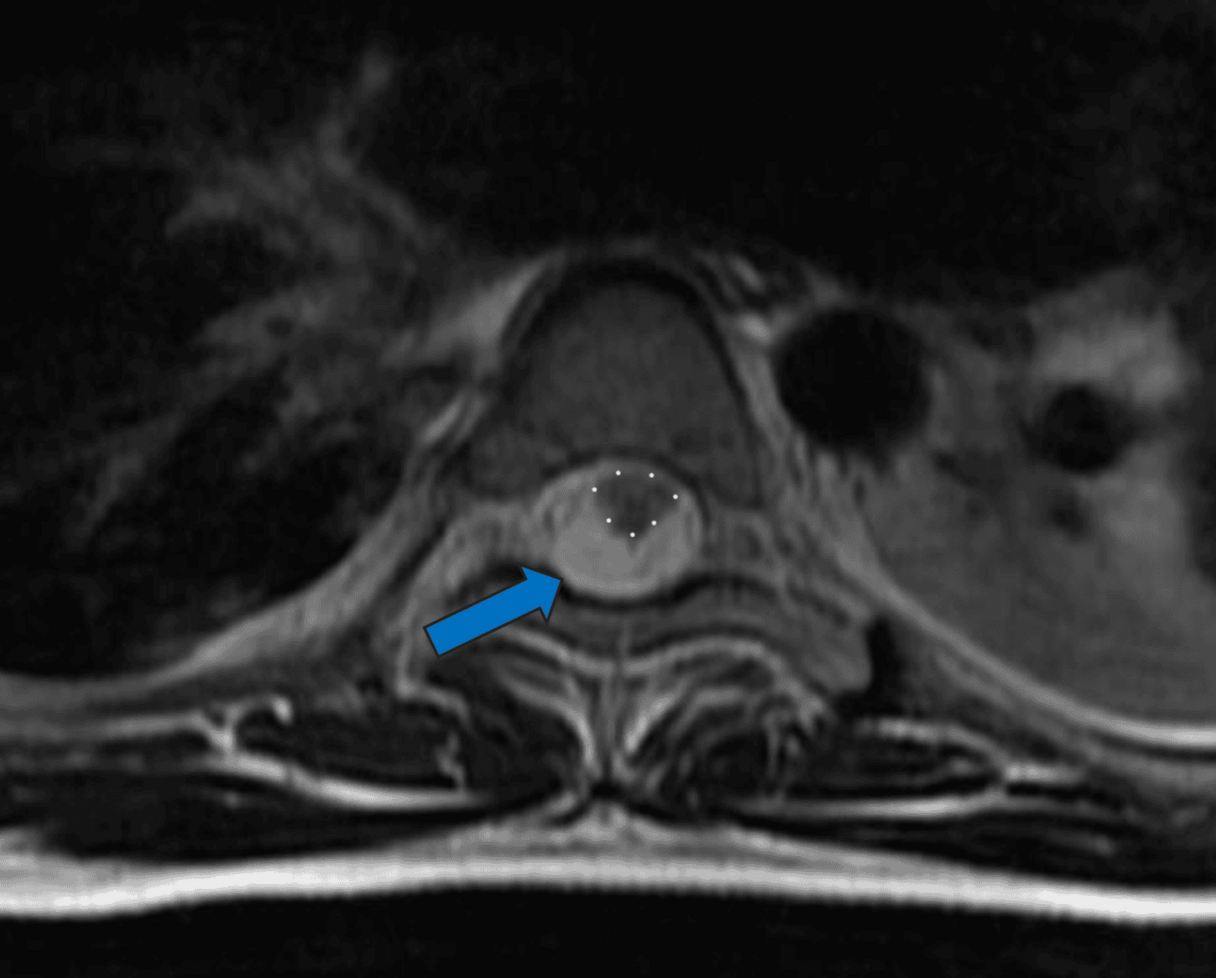
Axial T2 MRI showing metastasis (hyperintense area - blue arrow) compressing the thoracic spinal cord (hypointense area - outlined in white dots) again demonstrating disease progression with resulting lower and upper extremity weakness.

A non-contrast head CT revealed no acute changes compared to prior imaging. The pontine mass at the craniocervical junction remained stable, per an MRI of the brain. However, an MRI of the cervical, thoracic, and lumbar spine revealed extensive lobulated intradural lesions extending from the upper thoracic spine to the sacrum, causing significant mass effect and cord displacement, most notably at T1-T2 and T10-T11. The lesions encased the cauda equina and filled the lower lumbosacral spine from L3 to S2 level with associated mass effect on the cauda equina (Figures 3, 4).

**Figure 5:**
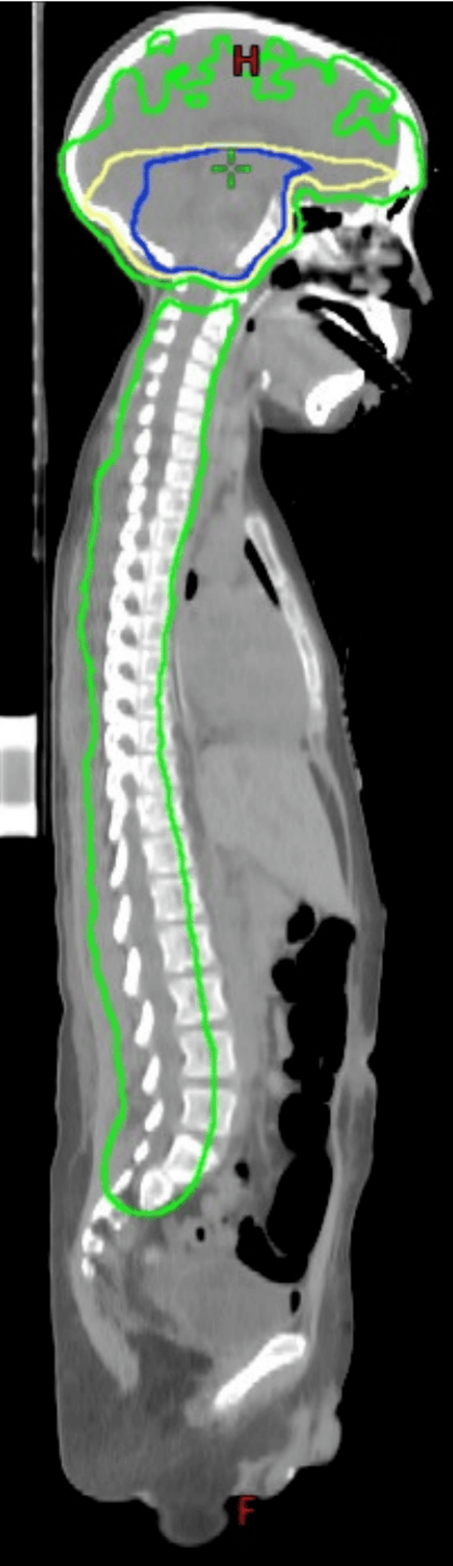
Sagittal view of craniospinal irradiation minimizing the dose to the brainstem. The 4000 cGy isodose line is green; the 5000 cGy isodose line is yellow; the 6000 cGy isodose line is dark blue

He underwent a course of salvage radiation therapy to the craniospinal axis following disease progression. Due to concerns of impending spinal cord compression, he initially received 800 cGy in four fractions targeting the lower cervical, thoracic, and lumbar spine to allow for immediate treatment initiation while the volumetric modulated arc therapy (VMAT) plan was being finalized (which required 2-4 days). VMAT is a radiation therapy technique using multiple arcs to treat the patient, which has been shown to have significant dosimetric advantages compared to conformal radiation therapy [6]. Once the VMAT plan was completed, the patient received an additional 3600 cGy in 18 fractions to the craniospinal axis, excluding the brainstem. To compensate for the initial exclusion of the upper cervical spine, this region was treated with a slightly higher dose of 220 cGy per fraction to ensure adequate coverage. The clinical target volume included the brain (excluding the brain stem) and the spinal cord, extending inferiorly to the filum terminale as noted on MRI. A PTV margin of 0.5 cm for the brain and 1 cm for the spinal cord was applied (Figure 5) [7]. 

The brain, with the dose limited to the brainstem, was also treated secondary to concerns of metastatic disease seeding the brain. The total dose was approximately 4400 cGy in 22 fractions to the PTV, which included the craniospinal axis, with the dose limited to the brainstem [6,8,9]. The decision to proceed with a definitive course of radiation therapy and not a palliative course was discussed in our pediatric tumor board. The decision to proceed with definitive radiation was to obtain long-term disease control. An MRI obtained near the completion of radiation therapy revealed a marked reduction in the size of the spinal lesions, with decreased mass effect on the cervical and thoracic spinal cord. The brain MRI remained stable. The patient was discharged from the PICU and did not require ventilation after five days of radiation therapy. However, he developed significant left upper extremity paralysis and partial right upper extremity weakness. He remained paraplegic below the trunk with bladder and bowel incontinence. His overall condition stabilized post-treatment, though with severe neurological deficits. He was discharged with home healthcare and underwent physical therapy.

A summary plan for the radiation therapy was generated, and the mean radiation dose to the brainstem was 6532 cGy. The spinal cord and optic nerves received a maximum dose of 4575 cGy and 5673 cGy, respectively. The dose to the kidneys was within tolerance, with less than 10% of the volume of the right and the left kidney combined receiving greater than 1800 cGy.

**Figure 6:**
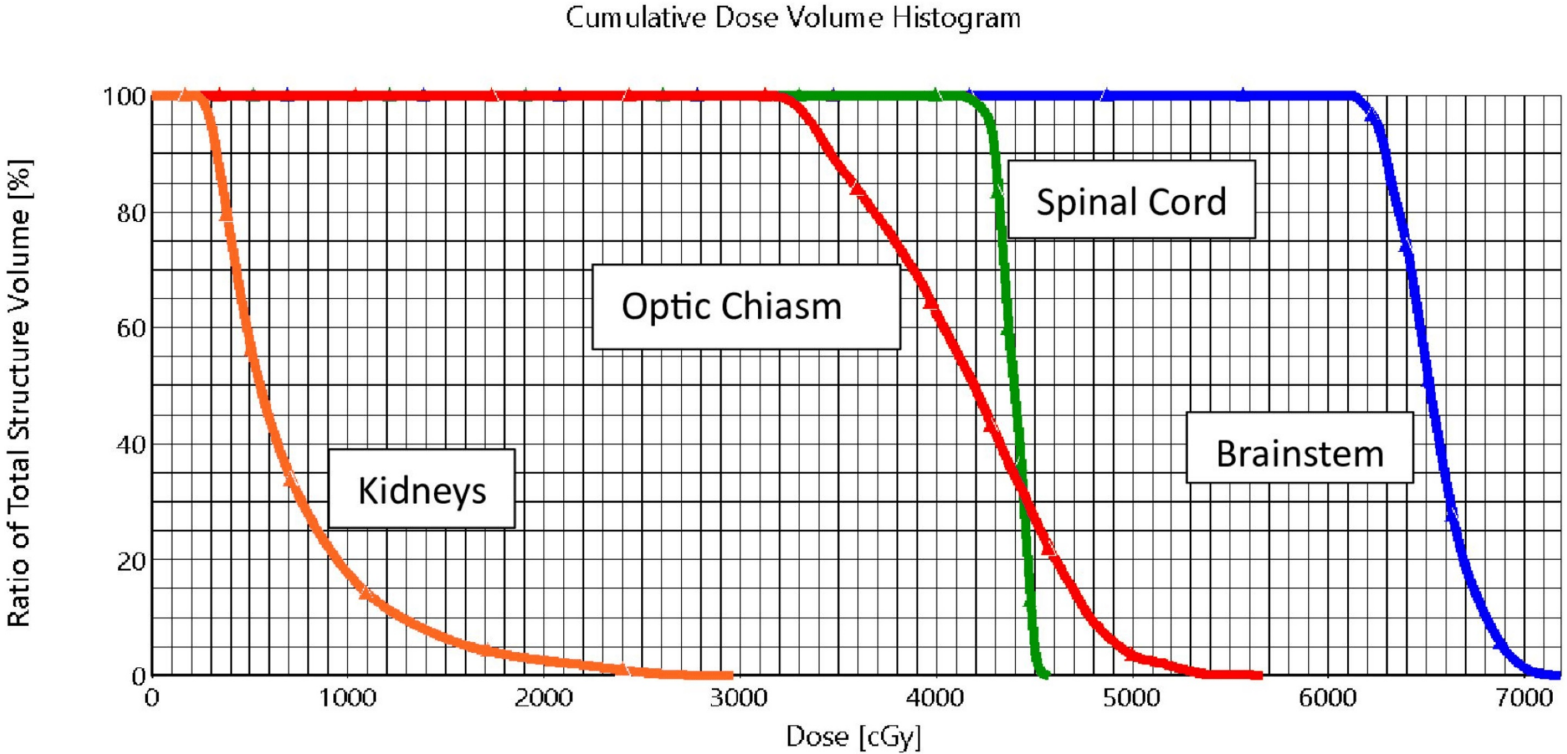
Dose volume histogram of the organs at risk. Blue indicates the brainstem; green indicates the spinal cord; red indicates the optic chiasm; orange indicates the kidneys

The following constraints were identified for the treatment of these patients. The maximum dose to the spinal cord (Dmax) was less than 4600 cGy; an attempt was made to keep the brainstem as low as possible and to keep the optic chiasm below 6000 cGy. The dose to the spinal cord was based on medulloblastoma craniospinal data in which the spinal cord in a patient with diffuse disease is limited to 4500 cGy, and the brain is treated with the same dose with a boost to the posterior fossa or the areas of disease to 5400 cGy [6,8,9]. Since the disease appeared not to have significantly progressed in the brainstem, the decision was made to keep the dose to the brainstem as low as possible. Secondary to concerns of long-term toxicity with this treatment, the dose to the spinal cord and the brainstem was planned as low as possible. A dose-volume histogram is displayed in Figure 6.

An MRI of the brain, cervical, thoracic, and lumbar spine obtained near the completion of radiation therapy revealed a marked reduction in the size of the spinal lesions, with decreased mass effect on the cervical and thoracic spinal cord. The brain MRI remained stable. The patient was discharged from the PICU and did not require ventilation after five days of radiation therapy. However, he developed significant left upper extremity paralysis and partial right upper extremity weakness. He remained paraplegic below the trunk with bladder and bowel incontinence. His overall condition stabilized post-treatment, though with severe neurological deficits. He was discharged with home healthcare and underwent physical therapy. 

**Figure 7:**
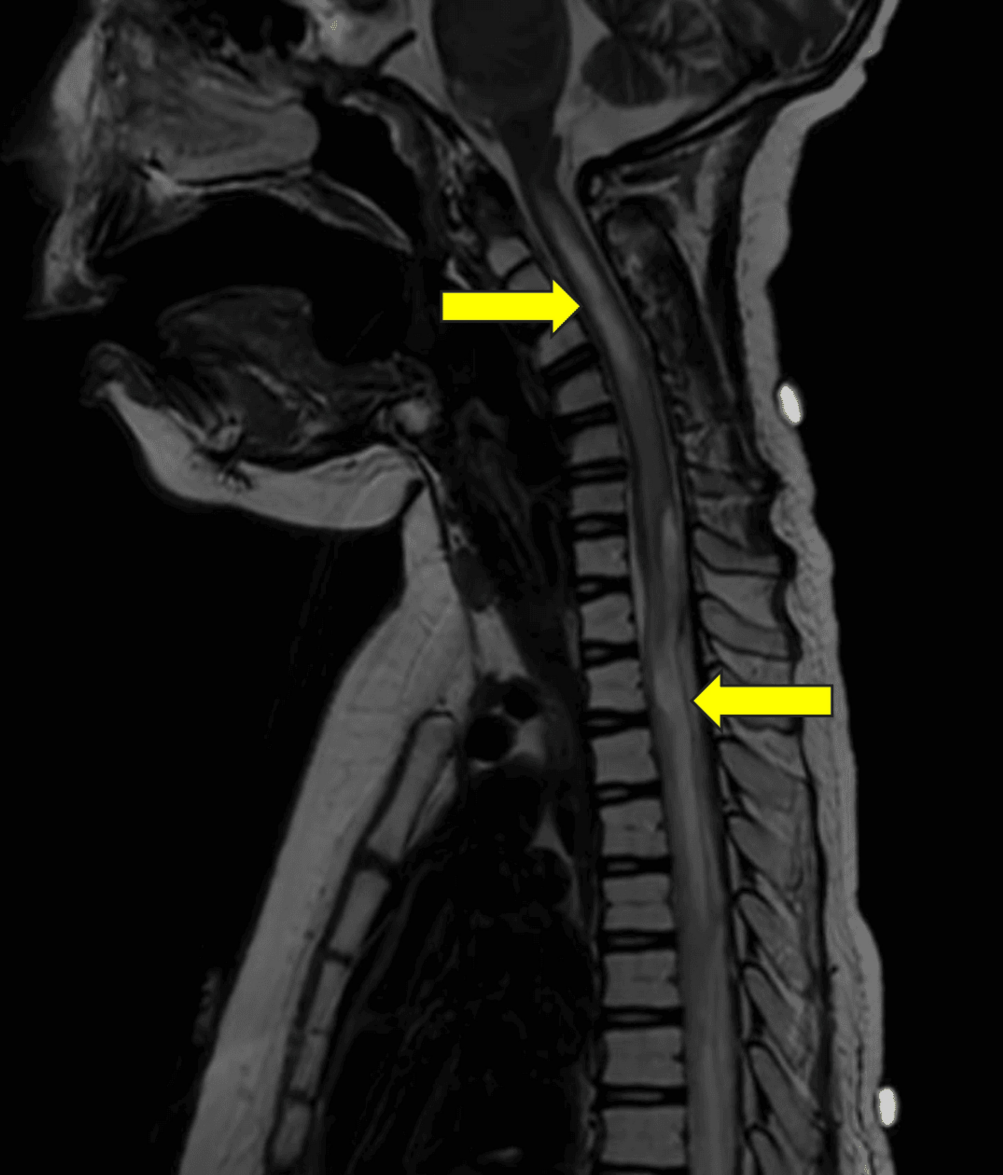
Sagittal T2 MRI showing tumor progression (yellow arrows)

Two months status post-treatment, his condition did not worsen clinically, and he had minimal improvement in his lower or upper extremity strength. A brain MRI showed that the pontine glioma appeared grossly stable in size with post-radiation changes. An MRI of the spine obtained two months after the completion of treatment revealed innumerable lobulated non-enhancing intradural lesions throughout the spine. Some of these lesions remained stable, and some had progressed in size (Figure 7).

He continued to decline neurologically and was placed in hospice. This case highlights the rare presentation of DIPG with extensive spinal metastases and underscores the importance of vigilant neurological monitoring and timely imaging in patients with progressive symptoms. A summary of his treatment is shown in Table 1.

**Table 1:** An outline of the time of presentation, symptom progression, results of MRI imaging, and treatment including the radiation dose and technique.

Time of Presentation	Symptom Progression	MRI Imaging Findings	Radiation Dose	Radiation Therapy Technique
Age 8 years – initial presentation	Slurring of speech, drooling from the mouth, and right-hand weakness	MRI brain -expansile 4.3 x 2.2 x 4.6 cm intra-axial mass lesion with an epicenter in the pons.	5040 cGy in 28 fractions to the brainstem lesion plus a 1 cm margin	Intensity modulated radiation therapy (IMRT)
18 months later, after the completion of radiation therapy to the brainstem lesion, Age 9 years.	Severe bilateral lower extremity pain, inability to ambulate, refusal to move his left lower extremity, abdominal pain, urinary incontinence, and constipation for over a week. Admitted to the PICU and intubated	MRI spine revealed extensive lobulated intradural lesions extending from the upper thoracic spine to the sacrum, causing significant mass effect and cord displacement. Brain MRI - pontine glioma appears grossly stable in size.	800 cGy in four fractions targeting the lower cervical, thoracic, and lumbar spine, and 3600 cGy in 18 fractions to the craniospinal axis, excluding the brainstem.	VMAT – Volumetric modulated arc therapy
Near the completion of radiation therapy 19 months after the initial presentation.	Extubated and discharged from the PICU. Patient with significant left upper extremity paralysis and partial right upper extremity weakness. Bladder and bowel incontinence.	MRI spine - marked reduction in the size of the spinal lesions, with decreased mass effect on the cervical and thoracic spinal cord. The brain MRI remained stable.	No additional treatment	No additional radiation treatment
Two months after the completion of radiation therapy to the craniospinal axis. Twenty-one months after the initial presentation.	Left upper extremity paralysis and partial right upper extremity weakness. Bladder and bowel incontinence.	MRI spine - innumerable lobulated non-enhancing intradural lesions throughout the spine. Some are stable, and some are slightly larger than before. Brain MRI - pontine glioma appears grossly stable in size with post-radiation changes.	No additional treatment	No additional treatment

## Discussion

DIPG is a highly aggressive pediatric brain tumor characterized by rapid progression and a poor prognosis. The H3K27M mutation, histone H3 gene in which lysine 27 (K27) is replaced by methionine (M), is present in nearly 80% of DIPG cases and has redefined its classification as a high-grade glioma. This mutation disrupts epigenetic dysregulation, leading to global epigenetic dysregulation and aggressive tumor behavior [1,10]. Patients diagnosed with DIPG and the H3K27M-mutant have a median survival of 10.1 to 14.4 months, with current therapies offering limited efficacy [3].

DIPG classically presents with progressive cranial nerve deficits, long tract signs, and cerebellar dysfunction, often evolving over weeks due to the tumor’s rapid growth [11]. In this case, the patient initially exhibited progressive lower extremity weakness, gait instability, and urinary incontinence, suggestive of spinal cord involvement. Spinal cord involvement is rare in DIPG but necessitates consideration in cases of worsening neurological symptoms. Formal neurological assessments, including cranial nerve examination and Karnofsky Performance Status (KPS) scoring, could provide further insights into functional decline.

Advanced imaging remains crucial for diagnosis, usually revealing hallmark features such as T1 hypo-intensity, T2 hyper-intensity, and diffuse pontine infiltration. In our case, initial imaging showed characteristic pontine involvement without distant metastases. However, subsequent MRI following clinical deterioration revealed spinal metastatic disease with enhancement along the spinal cord, indicative of leptomeningeal dissemination. A structured table summarizing imaging findings over time, including the lesion size, contrast enhancement, and diffusion-weighted imaging findings, would aid in objective disease tracking.

Histopathological characterization and molecular characterization are essential for refining diagnosis and guiding treatment. Molecular testing has further enhanced diagnostic precision by enabling the identification of key mutations, including H3K27M, ACVR1 (Activin A Receptor Type 1), and TPS53 (Tumor Protein p53), which contribute to DIPG’s aggressive phenotype [3]. While biopsies of the brainstem lesion were previously difficult to obtain, advancements in neurosurgical techniques now make this possible. The tissue obtained from the biopsy can also be used for molecular diagnostics and research [11].

Metastatic progression in DIPG, though rare, poses significant diagnostic and management challenges. Leptomeningeal dissemination is reported in 4-33% of cases, often linked to cerebrospinal fluid seeding and white matter tract migration [4]. In this case, the patient developed spinal metastases several months after initial treatment, highlighting the need for vigilant monitoring. 

A comparable case involved a seven-year-old female patient who developed leptomeningeal and subependymal dissemination. Six months after completing whole brain radiation therapy (5400 cGy in 35 fractions), she underwent a follow-up brain MRI, which indicated leptomeningeal and subependymal seeding, raising concerns for recurrent disease. She underwent a supraorbital keyhole craniotomy for frontal nodule biopsy with a right eyebrow incision. She unfortunately passed away due to cerebral edema and central herniation nine days after surgery. Histopathological evaluation of the biopsy specimen revealed moderately cytoplasmic tumor cells consistent with an anaplastic astrocytoma [4]. 

Another case report describes an eight-year-old girl diagnosed with a disseminated diffuse midline glioma involving the pons, thalamus, temporal lobes, and orbit, as well as the brachial plexus [12]. A spine MRI at presentation revealed a paraspinal mass at the cervical-thoracic junction extending into the brachial plexus and nodular enhancement of the lumbosacral nerve roots. The patient underwent craniospinal and right orbit proton radiation therapy, to a total dose of 5220 cGy in 29 fractions, and proton radiation to the brachial plexus, to a total dose of 4650 cGy in 24 fractions. The patient developed significant ascites and was found to have intraperitoneal metastases and was subsequently placed in hospice [12]. 

Radiation therapy remains the cornerstone of DIPG treatment, providing temporary symptom relief but no substantial survival benefit. Our patient initially received definitive IMRT with a total dose of 5040 cGy in 28 fractions to the brainstem. Upon disease progression, salvage craniospinal irradiation (CSI) was administered with an initial 800 cGy in four fractions for acute spinal cord compression, followed by 3600 cGy in 18 fractions using IMRT. This fractionation schedule was selected for logistical reasons to allow immediate treatment initiation while finalizing the IMRT plan.

The decision to use CSI over localized palliative radiation warrants further discussion. Treatment of DIPG remains challenging due to its eloquent location and resistance to therapies. Fractionated radiation therapy is the standard of care, providing temporary symptom relief but no significant survival benefit. Furthermore, systemic therapies for DIPG have largely been unsuccessful due to the BBB and tumor resistance mechanisms. 

Temozolomide, a standard chemotherapy agent for adult glioblastoma, failed to improve survival in a UK phase II trial, combining it with radiotherapy in pediatric DIPG patients [13]. In our case, systemic therapy was not administered, though emerging treatments such as H3K27M-targeted therapies, CAR-T cell therapy, and convection-enhanced delivery techniques are under investigation [3,14]. Discussing this patient's potential experimental or off-label therapeutic options would add to the treatment landscape.

Experimental treatment methods like focused ultrasound and convection-enhanced delivery also show potential for improving therapeutic outcomes, though further research is needed.

Metastatic DIPG has profound implications for prognosis and treatment strategies. Metastatic progression worsens symptom burden and limits therapeutic options. The patient’s overall survival following salvage therapy should be reported and contextualized with existing data on metastatic DIPG. This case highlights the need for vigilance in monitoring spinal and leptomeningeal involvement, necessitating adaptations in diagnostic practices to ensure early detection. Enhanced imaging surveillance, including spinal MRI at regular intervals, may improve early detection of dissemination.

The rarity of metastatic DIPG highlights the importance of advancing our understanding of the disease’s pathophysiology, including tumor biology, microenvironmental factors, and molecular markers such as the H3K27M mutation, which may drive atypical behaviors like enhanced cell migration and invasion. Clinical trials targeting these molecular pathways provide hope for novel therapies, though their efficacy in metastatic cases remains uncertain. By advancing diagnostic approaches and exploring innovative treatments, we aim to improve outcomes for patients facing this devastating disease.

## Conclusions

DIPG remains a devastating pediatric malignancy with limited treatment options, particularly in metastatic cases. This case highlights the challenges of disease progression, treatment decision-making, and prognosis. Advancing our understanding of DIPG biology, optimizing radiation strategies, and exploring novel systemic therapies are critical steps toward improving outcomes for patients with this aggressive disease.
